# A Harmonic Linear Dynamical System for Prominent ECG Feature Extraction

**DOI:** 10.1155/2014/761536

**Published:** 2014-02-26

**Authors:** Ngoc Anh Nguyen Thi, Hyung-Jeong Yang, SunHee Kim, Luu Ngoc Do

**Affiliations:** ^1^Department of Computer Science, Chonnam National University, Gwangju 500-757, Republic of Korea; ^2^Department of Computer Science, Carnegie Mellon University, Pittsburgh, PA 15213, USA

## Abstract

Unsupervised mining of electrocardiography (ECG) time series is a crucial task in biomedical applications. To have efficiency of the clustering results, the prominent features extracted from preprocessing analysis on multiple ECG time series need to be investigated. In this paper, a Harmonic Linear Dynamical System is applied to discover vital prominent features via mining the evolving hidden dynamics and correlations in ECG time series. The discovery of the comprehensible and interpretable features of the proposed feature extraction methodology effectively represents the accuracy and the reliability of clustering results. Particularly, the empirical evaluation results of the proposed method demonstrate the improved performance of clustering compared to the previous main stream feature extraction approaches for ECG time series clustering tasks. Furthermore, the experimental results on real-world datasets show scalability with linear computation time to the duration of the time series.

## 1. Introduction

Clustering multiple time series data have received considerable attention in recent years in various applications, such as industries of finance, business, science domains, and medicine [1–8]. Clustering in time series is the unsupervised mining of grouping similar time series into a same cluster. Specifically, clustering finds groups of homogenous patterns into a cluster so that these patterns within the cluster bear strong similarity to other ones and dissimilarity to patterns in other clusters [9, 10].

Since the quality of clustering results relies strongly on good features extracted from multiple time series, a very important processing step is to identify compact features extracted from multiple coevolving time series. This step can be used to not only convert a series of original values to more meaningful information, such as more understandable and interpretable information, but it also can lead time series to a lower dimensionality with the most relevant features.

This paper is motivated by mining these essential features of the medicine applications, namely, electrocardiography (ECG) time series. The problems are studied based on the challenges across time series applications such as time shifts effects, nearby frequencies, and harmonics. By exploiting the temporal evolving trends and the correlation characteristics of coevolving time series, meaningful features which help to achieve the best clustering accuracy can be captured.

Feature extraction can efficiently describe time series since suitable representations reduce the feature spaces. This provides highly efficient features for knowledge discovery so that they help to improve performance as well as the speed of the mining algorithms.

Many time series representations have been proposed to extract features. The well-known dimension reduction approaches, namely, Principal Component Analysis (PCA) and Singular Value Decomposition (SVD), are powerful tools to discover linear correlations across multiple time series [11–15]. They effectively give the optimal low-rank approximation of the dataset by removing the variables with lower energy [14]. However, these methods ignore time-evolving patterns since their characteristics are not designed for tracking the ordering of the rows or the columns. Moreover, the projection of the multiple time series into low-dimensional principal components is not easy to interpret. They do not clearly exhibit the characteristic of each pattern. As a result, applying clustering methodology with these reduction methods to multiple time series leads to poor performance.

One of the popular alternative approaches for feature extraction on multiple time series is Discrete Fourier Transform (DFT), where the original time series data is projected into the frequency domain [16–18]. The main advantage of DFT is to capture frequencies in a single time sequence. However, DFT lacks the dynamics; hence, clustering based on Fourier coefficients is not unsuitable. Another method based on Linear Predictive Coding Cepstrum (LPCC) which also provides distinguished features in time series is well known in signal recognition [8, 19, 20]. It is efficient for clustering performance by means of a few coefficients. However, the representations of achieved LPC cepstrum features are hard to interpret.

The Linear Dynamical System, known as Kalman filters, has been commonly used for time series analysis because of its simple implementation and extensibility [21–23]. Kalman filter can capture correlations among multiple time series and learn their evolving dynamics. However, the time shift effects, which are temporal gaps between two time series, cannot be handled. The representations of the extracted features are not also clear. The resulting model parameters thus lead to poor clustering performance.

Another popular feature extraction method, known as Dynamic Time Warping (DTW), can handle time shifts across the sequences. However, this approach ignores temporal dynamics. Therefore, applying DTW directly for clustering purpose cannot give good results [24].

Autoregression Moving Average (ARMA) is also a method for feature extraction. However, ARMA parameters do not provide a reliable method since different sets of parameters can be obtained even from time series with similar structures. As a result, the clustering performances will be affected dramatically [25, 26].

In this paper, we applied an approach of prominent feature extraction based on a Harmonic Linear Dynamical System (HLDS) [21] for unsupervised learning of time series in biomedical applications, specifically in ECG. Since the applications have characteristics of lag correlation and temporal dynamics, HLDS works well for the prominent feature extraction of the time series in the same period with different lags and light amplitudes. In addition, HLDS is expected to identify periodic patterns and group them into one cluster. These series of frequencies are known as harmonic series which contain several mixtures of frequencies, especially in sensor measurements, such as in human voice and in human motion domain [23, 27].

Our prime objective is to exploit two common characteristics of multiple coevolving time series: correlations and temporal dynamics for meaningful feature extraction. Correlation reflects the relationships among multiple time sequences, while dynamic property discovers the temporal moving trends of multiple time series by automatically identifying a few hidden variables. For example, a particular medical signal of physiological records in ECG application characterizes a specific symptom of a patient such as a malignant ventricular arrhythmia person. Therefore, each time series differs from the others in dynamics since time series encodes temporal dynamics along the time ticks. By capturing correlations, we can achieve good interpretable features in the presence of time shift effects and small shift in frequency. By exploiting the evolving temporal components, we can find the clusters of time series by grouping them with similar temporal patterns.

In order to evaluate the effectiveness of the applied method considering clustering accuracy, reliability, and complexity aspects, this paper demonstrates the clustering results of ECG time series using *k*-means algorithm. The method we applied discovers useful and understandable features from time series for clustering purposes. Moreover, its computational time scales up to the duration of the time sequences. We demonstrate comparative study to assess the performance of the proposed method against previous feature extraction algorithms such as PCA [11, 12], DFT [17, 18], original Kalman filter [22, 23], and LPCC [19, 20].

The rest of the paper is organized as follows. Background of the underlying Linear Dynamical System theory and the proposed model setup are given in the upcoming Section 2. In Section 3, the experimental time series dataset of ECG used in this paper are illustrated and the empirical evaluations have been conducted to assess effectiveness of the proposed feature extraction method in clustering multiple time series. Finally, we present our conclusions in Section 4.

## 2. Materials and Methods

### 2.1. Fundamentals of Linear Dynamical System

Multidimensional time series dataset *Y* is an ordered sequence of data points measured at equal time intervals, denoted by *Y* = {*y*_1_, *y*_2_, *y*_3_,…, *y*_*T*_}, where each vector *y*_1_, *y*_2_, *y*_3_,…, *y*_*T*_ is formed of *M* observations recorded at time ticks *t* = 1, *t* = 2,…, *t* = *T*, respectively. Time series collections can be formed as the matrix below:

(1) $$\begin{array}{c} Y=\left[\begin{array}{cccccc} y_{1}^{1} & y_{1}^{2} & \ldots & y_{1}^{i} & \ldots & y_{1}^{M}\\ y_{2}^{1} & y_{2}^{2} & \ldots & y_{2}^{i} & \ldots & y_{2}^{M}\\ \vdots & \vdots & & \vdots & & \vdots \\ y_{t}^{1} & y_{t}^{2} & \ldots & y_{t}^{i} & \ldots & y_{t}^{M}\\ \vdots & \vdots & & \vdots & & \vdots \\ y_{T}^{1} & y_{T}^{2} & \ldots & y_{T}^{i} & \ldots & y_{T}^{M} \end{array}\right]. \end{array}$$

In particular, each row vector includes all of the observations for one certain time tick which is an ordered sequence of *T* vectors each having dimensionality of *M*. Each column denotes the observations for one particular measurement, which is an unordered collection of *M* one-dimensional time series vector [1, 2].

Linear Dynamical System (LDS), known as Kalman filter, has been used to model multidimensional time series data. By taking the definition of time series above as a matrix for a dynamical system, this means that multidimensional time series data can be presented by a matrix *Y*_*M*×*T*_ of the variables *M* and observed time ticks *T*. LDS builds a statistical model to represent the state of the hidden variables which are evolving to a linear transformation leading to the observed numerical time sequences. LDS captures the correlations among multiple signals by means of choosing a proper number of hidden variables so that the model can learn the dynamics of time series data [13, 14, 28]. LDS for a multidimensional time sequence is modeled by the following equations:

(2) $$\begin{array}{c} z_{1}=\mu_{0}+\omega_{0} \end{array}$$

(3) $$\begin{array}{c} z_{n+1}=A\cdot z_{n}+\omega_{n} \end{array}$$

(4) $$\begin{array}{c} y_{n}=C\cdot z_{n}+\varepsilon_{n}, \end{array}$$

where *θ* = {*μ*_0_, *Q*_0_, *A*, *Q*, *C*, *R*} is the set of parameters. Vectors *y*_*n*_ and *z*_*n*_ denote observed data sequences and hidden variables at time *n*, respectively. *μ*_0_ is an initial state for hidden variables of the whole system. The transition dynamic matrix *A* relates to the transition of the state from the current time tick to the next time tick with noise {*ω*_*n*_}. The matrix *C* is the observation projection with noise {*ε*_*n*_} at each time *t*. All noise *ω*_0_,  *ω*_*i*_, and *ε*_*i*_  (*i* = 1,…, *T*) are zero-mean and normally distributed random variables with covariance matrices *Q*_0_, *Q*, and *R*, respectively [29]. The Expectation Maximization (EM) algorithm is utilized to learn the component models and estimate hidden variables [30]. In the model, each row of output matrix *C* corresponds to one sequence and, therefore, can be used as features in clustering. However, the clustering quality is not good since these features cannot provide high interpretability excluding information of time shift. Time shift effect or phase shift indicates that two time series have the same frequency but different phase lags.

### 2.2. Proposed Feature Extraction Methodology

In this section, the proposed method for ECG dataset is set up to illustrate how to exploit the interpretability of prominent features extracted from multiple time series in order to improve the clustering quality. Since each row of output transition matrix *C* of the LDS model does not present distinct characteristic of the corresponding series, we explore further expecting to discover deeper hidden patterns for each sequence of LDS by learning the straight forward transition matrix *A* and output projection matrix *C*.

First of all, the hidden dynamics are learned via Linear Dynamical System (LDS), capturing the series of hidden variables which are evolving according to the linear transformation *A*; they are linearly transformed to the observed numerical sequences [13, 28]. This implies that the series of hidden variables, *z*_*n*_, are evolving over time ticks with linear transition matrix *A*. Also, the observed data sequences, *y*_*n*_, are generated from these series of hidden variables with a linear output projection matrix *C* [21].

Secondly, after achieving the hidden variables from the LDS system, the canonical form of the hidden variables is identified. However, these hidden variables are hard to interpret since they are mixed in the observation sequences. Therefore, we need to make them compact and uniquely identify. Equation (3) represents the hidden variables depending on the eigenvalues of the transition matrix *A* [21]. Those eigenvalues can capture the amplitude and frequencies of the underlying signals of hidden variables which are referred to as harmonics. As a result, normalizing the transition matrix can directly reveal the amplitude, frequency, and the mixtures of the given set of sequences. Harmonic Linear Dynamical System (HLDS) uses eigendecomposition on transition matrix *A* in order to find harmonics as well as the mixing weight of harmonics. The eigendecomposition corresponds to the diagonal matrix (Λ) of eigendynamics and eigenvector (*V*) of *A* as follows:

(5) $$\begin{array}{c} A=V\Lambda V^{*}. \end{array}$$

In LDS, the output projection matrix *C* illustrates how the hidden variables are translated into observation sequences with linear combinations. Therefore, we need to compensate *C* matrix to achieve the harmonic mixing matrix *C*_*h*_ in order to obtain the same observation sequences from eigendynamics (Λ) as the transition matrix:

(6) $$\begin{array}{c} C_{h}=C\cdot V. \end{array}$$

The canonical hidden variables will be

(7) $$\begin{array}{c} \mu_{0}^{\text{new}}=V^{*}\cdot \mu_{0},\\ z_{n}^{\text{new}}=V^{*}\cdot z_{n}. \end{array}$$

*V* contains conjugate pairs of columns corresponding to the conjugate pairs of eigenvalues in Λ. Therefore, the harmonic mixing matrix *C*_*h*_ must contain conjugate pair of columns corresponding to the conjugate pairs of the eigenvalues in Λ:

(8) $$\begin{array}{c} {\;\;\;z}_{n}^{\text{new}}=\Lambda^{n-1}\cdot \mu_{0}^{\text{new}}+\text{noise},\\ \;\;y_{\text{new}}={C_{h}\cdot \Lambda }^{n-1}\cdot \mu_{0}^{\text{new}}+\text{noise}. \end{array}$$

From the equation, we can obtain all hidden variables *z*_*n*_, canonical hidden variables *z*_*n*_^new^, and observation *y*_*n*_. They are mixtures of a set of scaling such as growing, shrinking, or stationary sinusoid signals of data-dependent frequencies which are referred to as harmonics [21]. Their characteristics of frequencies and amplitudes are completely defined by the eigenvalues of the transition matrix *A*.

Thirdly, the harmonic mixing matrix *C*_*h*_ is calculated in the second step. From here, the contribution of each harmonic to the resulting observation sequences is found. This means that each row of *C*_*h*_ represents the characteristic of each sequence in the domain of the harmonics and thus, it can be used to cluster sequences. However, the harmonic mixing matrix will fail to group similar sequences with phase shifts or time shifts because it tells not only the strength of each eigen dynamic, but also encodes the required phases for different sequences. To eliminate the phase/lag effect by taking the magnitude of the harmonic mixing matrix *C*_*h*_, we will obtain the same column for the conjugate column of *C*_*h*_. By dropping these duplicated columns, we will obtain the harmonic magnitude matrix *C*_*m*_, which tells how strong each harmonic base participates in the observation time sequences and solves lag challenge as well.

Lastly, in order to obtain the interpreted features for each sequence, we apply the dimension reduction approach with SVD on the harmonic magnitude matrix, ${\tilde{C}}_{m}\approx U_{k}\cdot S_{k}\cdot V_{k}^{T}$, where ${\tilde{C}}_{m}$ is the column centered from *C*_*m*_, *U*_*k*_ and *V*_*k*_ are orthogonal matrices with *k* columns, and *S*_*k*_ is a diagonal matrix. The interpretability of the prominent features can be obtained as *U*_*k*_ · *S*_*k*_. For the proper number of hidden dimension *h* in our ECG application, we use Fukunaka's principle rule [15, 21]. We choose *h* as the one with the 98th percentile of the total sum of squared singular values with *s*_*i*_'s being the singular values of *Y* in descending order. The formula is as follows:

(9) $$\begin{array}{c} h⟵\underset{h}{\arg}\frac{\sum_{j=1}^{h}s_{j}^{2}}{\sum_{i=1}^{m}s_{i}^{2}}. \end{array}$$

In summary, HLDS includes four steps: (1) learning hidden variables using LDS, (2) taking eigendecomposition on transition matrix *A* to find the canonical form of hidden variables, which helps to find harmonics and mixing weight of harmonics, (3) taking the magnitude of the harmonic mixing matrix to eliminate phase shift, and (4) using SVD to combine harmonics.

## 3. Experimental Results and Discussion

In order to show the validation of the clustering by HLDS, we carry out experiments on real ECG dataset taken from PhysioNet http://www.physionet.org/physiobank/database/ [31, 32]. A Harmonic Linear Dynamical System for the prominent feature extraction of ECG application is investigated successfully since the applications have the characteristics of lag correlations and temporal dynamics. These extracted features can group time series patterns in the same period with different lags and light amplitudes. Moreover, the applied method is expected to identify periodic patterns and group them into one cluster. ECG dataset contains three different groups of ECG time series: 13 time series of ECG recordings of healthy people, 22 time series of people having malignant ventricular arrhythmia, and 30 time series of people having supraventricular arrhythmia, taken from the following specific links:

- MIT-BIH Healthy/Normal Sinus Rhythm Database http://www.physionet.org/physiobank/database/ nsrdb/;
- MIT-BIH Malignant Ventricular Arrhythmia Database http://www.physionet. org/physiobank/database/vfdb/;
- MIT-BIH Supraventricular Arrhythmia Database http://www.physionet.org/physiobank/database/ svdb/.

There are two collections which are investigated. Collection 1 contains a group of healthy people and malignant ventricular arrhythmia while collection 2 is obtained by the group of healthy and supraventricular arrhythmia people. To evaluate the effectiveness of the feature extraction for the clustering, both the quality and scalability of normalized time series are considered against previous feature extraction approaches such as LPCC, PCA, DFT, and original Kalman filter. Normalization is carried out to compensate the differences in level and scale of dataset to a zero-mean and unit variance. In the experiment, we use the first two coefficients and cluster them by *k*-means algorithm with Euclidean distance. For choosing the proper number of hidden variables, we set 98% of energy in each original data which is corresponding to the hidden dimension of 20 and 35 for collection 1 and collection 2, respectively.

To evaluate the quality of the clustering results, we use the confusion matrix since we know the ground truth labels of each sequence. The clustering performance of different methods on collection 1 of real ECG time series is recorded as follows: the proposed method (94.29%), LPCC (85.71%), KF (42.9%), DFT (57.14%), and PCA (40%). Compared to the previous feature extraction methods, the average performance of applied HLDS on real ECG datasets demonstrates significant performances, that is, 9.1%, 54.5%, 39.39%, and 57.57% clustering improvement against the LPCC, original Kalman filter, DFT, and PCA, respectively.

Since this method can discover deeper hidden patterns which can capture correlation and temporal dynamics successfully, it provides the group of distinct harmonics that helps to handle the presence of the time shift effect with small shifts in frequency. These harmonic groups represent good resulting features which lead to good clustering as well as visualization.

**Figure 1:**
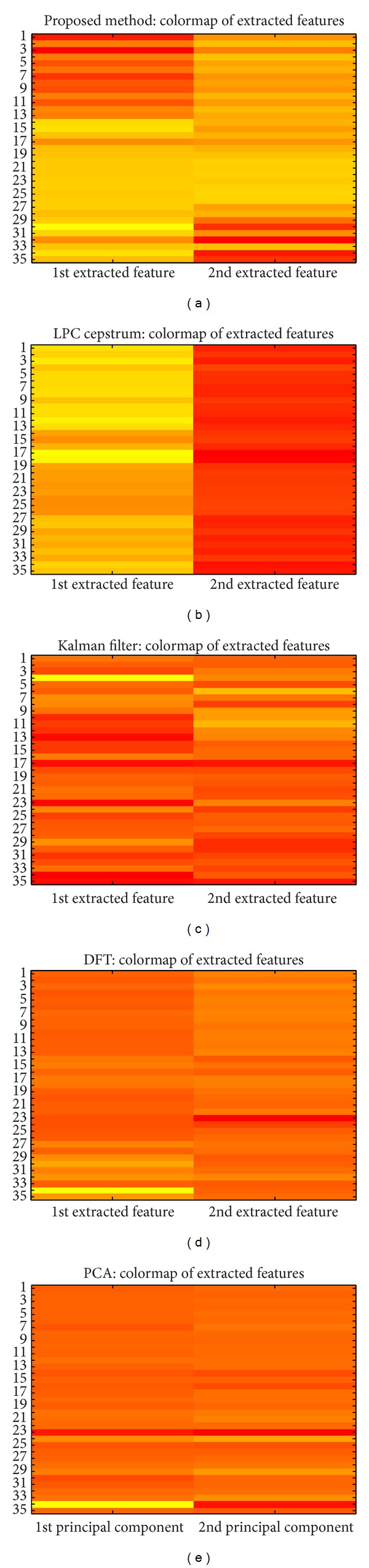
The first two extracted features of algorithms on EEG dataset.

In more detail, Figure 1 shows the comparison of the first two extracted features of ECG dataset by the proposed method, LPCC, original Kalman filter, DFT, and PCA. In each heat map of Figure 1, the rows represent the number of sequences of dataset and the columns show the first two features extracted from each method. Only the proposed method clearly illustrates the characteristic of the two clusters, normal and malignant ventricular arrhythmia especially in the 1st extracted features in Figure 1(a), where the rows have similar feature values colored in orange and yellow, so that they are grouped in the same cluster. On the other hand, the four remaining methods, Figures 1(b), 1(c), 1(d), and 1(e), do not show meaningful interpreted features.

The proposed method gives wrong clustering results for the 17th and 32nd signals. Even though the 17th and 32nd signals are malignant ventricular arrhythmia signals actually, the shapes of these signals are very similar to the normal case. Therefore, when applying method, they discover similar features as the normal cases; consequently, they are clustered to the normal cluster.

**Figure 2:**
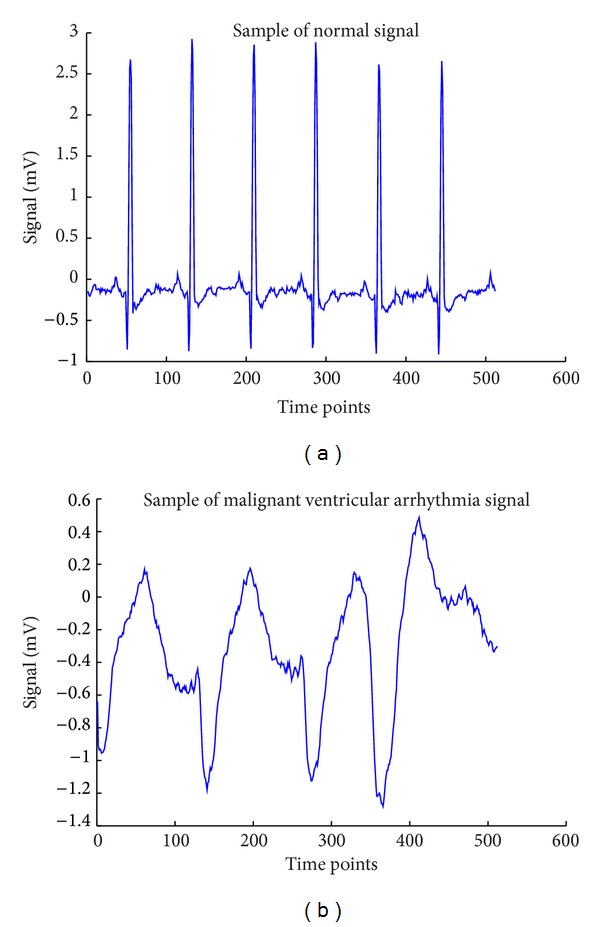
Original samples of normal and malignant ventricular arrhythmia time series, respectively.

**Figure 3:**
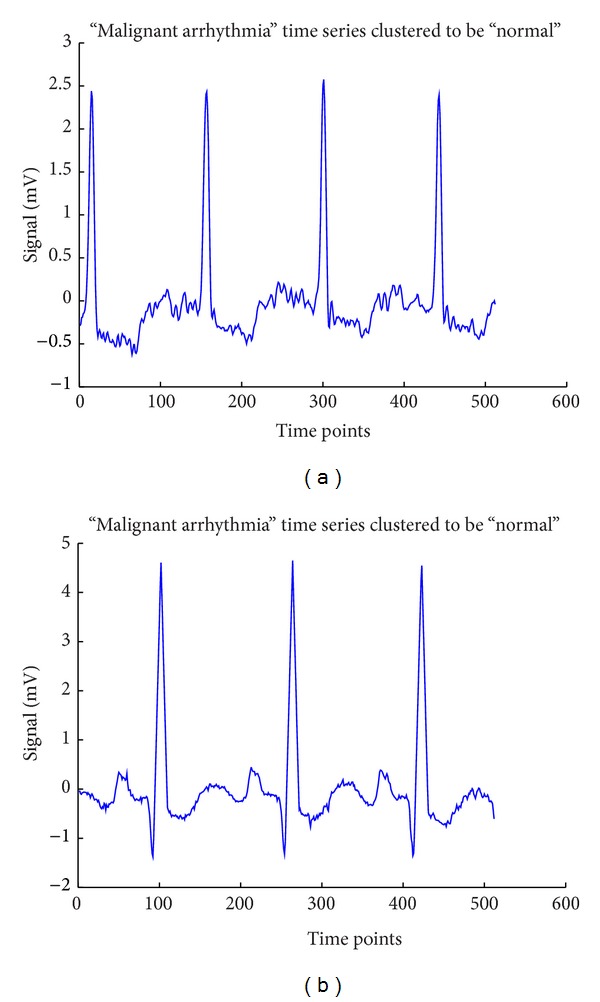
The 17th and 32nd malignant ventricular arrhythmia patterns clustered to be wrong cluster.

To verify them, the 17th and 32nd time sequences of Figure 1(a) are plotted in Figure 2 where the patterns of 17th and 32nd from Figure 1 are in very similar shape compared to the normal signal shown in Figure 3(a) where Figure 3(b) is a sample signal of malignant ventricular arrhythmia case.

**Figure 4:**
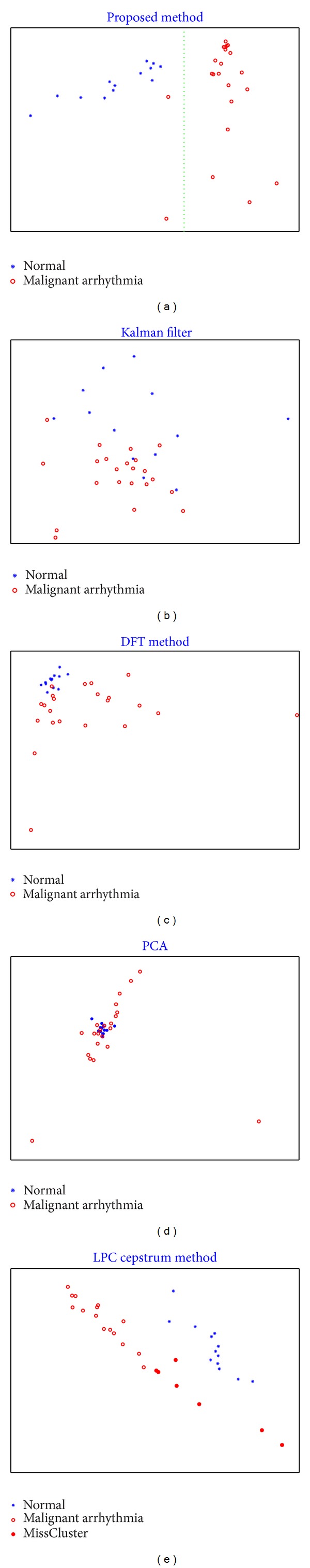
Scatter plot of clustering visualization.

Figure 4 shows the scatter plots of the first feature and the second feature from different methods for ECG clustering. On the other hand, the first two of extracted features are plotted to evaluate how much these features contribute to ECG recognition. It is observed that the HLDS shows clear separation of dots compared to the other approaches.

The proposed method again performs the best clustering accuracy for collection 2 which consists of healthy and supraventricular arrhythmia persons. The error rates for all of experimental methods are shown as follows: proposed method (0.139), LPCC (0.2326), KF (0.4419), PCA (0.3256), and DFT (0.3953).

**Figure 5:**
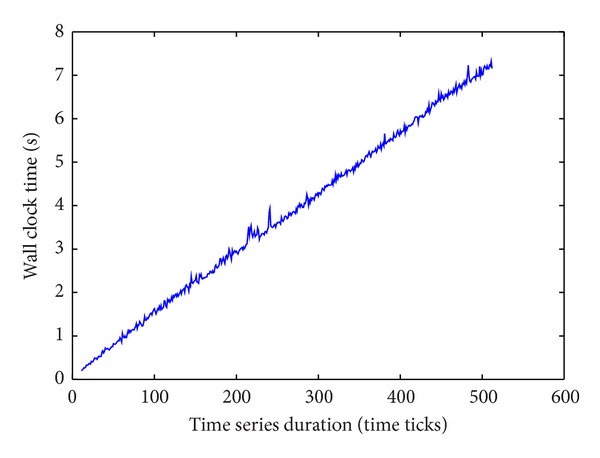
Linear execution time with respect to the length of sequences.

The computational complexity of the HLDS is shown in Figure 5, executed on collection 1. It can be observed that wall clock times lie on almost a straight line over the duration of sequences since it would improve the scalability in ECG dataset. The computation time of HLDS is satisfied when the time duration *T* of the time sequences is increased.

## 4. Conclusions

The proposed method, HLDS, considers the problem of handling the challenges of time series, namely, time shift, nearby frequencies, and harmonics. The applied method demonstrates the efficiency for solving these challenges in real applications of ECG time series domain. Interpretability of prominent features was discovered for the clustering as well as visualization. In most cases, HLDS gives the best result compared to the other feature extraction techniques such as Kalman filter, LPC cepstrum, DFT, and PCA. Moreover, the performance results show almost a linear speedup as we increase the input of the dataset.

For further study, we will investigate the harmonic linear dynamical system over much longer time series with missing values in various applications.
